# Functional Gait Can Be Affected by Noise: Effects of Age and Cognitive Function: A Pilot Study

**DOI:** 10.3389/fneur.2021.634395

**Published:** 2021-02-09

**Authors:** Margot Buyle, Viktoria Azoidou, Marousa Pavlou, Vincent Van Rompaey, Doris-Eva Bamiou

**Affiliations:** ^1^Experimental Laboratory of Translational Neuroscience, Faculty of Medicine and Health Sciences, University of Antwerp, Antwerp, Belgium; ^2^Faculty of Life Sciences & Medicine, Centre for Human & Applied Physiological Sciences, King's College London, London, United Kingdom; ^3^Department of Otorhinolaryngology and Head & Neck Surgery, Antwerp University Hospital, Edegem, Belgium; ^4^Faculty of Brain Sciences, University College London (UCL) Ear Institute, University College London, London, United Kingdom; ^5^Biomedical Research Centre, National Institute for Health Research, London, United Kingdom

**Keywords:** functional gait, cognition, hearing loss, passive listening, attention

## Abstract

**Background:** The ageing process may degrade an individual's balance control, hearing capacity, and cognitive function. Older adults perform worse on simultaneously executed balance and secondary tasks (i.e., dual-task performance) than younger adults and may be more vulnerable to auditory distraction.

**Aim:** The purpose of this study was to determine the effect of passive listening on functional gait in healthy older vs. younger adults, and to investigate the effect of age, functional gait, hearing ability and cognitive functioning on dual-task performance.

**Methods:** Twenty young and 20 older healthy adults were recruited. Functional gait (Functional Gait Assessment in silent and noisy condition), hearing function (audiogram; Speech in Babble test), and cognitive ability (Cambridge Neuropsychological Test Automated Battery) were measured.

**Results:** Overall, a significant difference between functional gait performance in silent vs. noisy conditions was found (*p* = 0.022), with no significant difference in dual-task cost between the two groups (*p* = 0.11). Correlations were found between increasing age, worse functional gait performance, poorer hearing capacity and lower performance on cognitive function tasks. Interestingly, worse performance on attention tasks appeared to be associated with a worse functional gait performance in the noisy condition.

**Conclusion:** Passive listening to multi-talker babble noise can affect functional gait in both young and older adults. This effect could result from the cognitive load of the babble noise, due to the engagement of attention networks by the unattended speech.

## Introduction

Balance control is not just an automatic process but also a perceptual motor task that requires cognitive function, especially attention (1). Ageing can be associated with a decline in balance, hearing, and cognitive function. Age-related changes in pathways that underpin balance control may lead to balance impairment or increased falls risk (2). There is, also, evidence that both age-related hearing loss [(3) for a review (4)] and age-related cognitive decline [(5, 6) for a review (7)] increase these risks. Furthermore, hearing loss is a potential risk factor for cognitive deficits in older adults [(8) for a review (9)]. In particular attention, working memory and executive function seem to be impaired in individuals with hearing loss [(9) for a review].

It is suggested that an inability to allocate attention could be an important factor contributing to balance constraints during gait in fallers (10). Older adults in particular need more attentional resources to keep their postural stability (10, 11), especially when multitasking (10). If the attention capacity is exceeded when performing two tasks together, a dual-task interference effect will be seen, i.e., the performance of one or both tasks deteriorates (12, 13).

There are several studies assessing dual-task effects on dynamic balance performance. Some of these have investigated the effect of auditory tasks on balance ability, but only three studies reported the effect of an attentional demanding “active” listening task on balance performance. Bruce and colleagues examined dual-task costs (i.e., the performance decrement that results from executing two tasks simultaneously) on a working memory task, and on balance recovery tasks in quiet and background multi-talker babble noise conditions. There was no effect of auditory challenge on postural measures for either young or old adults, as well as old adults with age-related hearing loss (14). An earlier study by Springer et al. (15) studied gait under dual tasking in young adults, old non-fallers and elderly at risk of falling. Gait was evaluated as a single task and under three different dual-task conditions (i.e., two active listening tasks and an arithmetic task). The performance of the tasks that required attention had a destabilising effect on the postural control of older fallers. However, they failed to find an age-associated increase in the dual-task effect on gait variability between non-fallers and young adults. Notably, all three groups showed a reduction in gait speed during all three dual-tasking conditions. Young adults possibly decreased their gait speed to remain stable, while older non-fallers decreased their speed and swing times (15). In another study, young adults and old adults had to listen to and report key words from a target sentence while walking. The researchers reported that walking required more cognitive resources for older than for younger adults, and proposed that effortful listening in elderly resulted in a competition for cognitive capacity required for walking (16).

Passive listening tasks might be particularly relevant to investigate since we live in a very noisy world (17). This may be especially interesting in older adults, as various neuroimaging studies have indicated their vulnerability to auditory distraction (18, 19). Stevens and colleagues found that the noisy fMRI environment induced an age-related distraction effect that was attributed to a misallocation of attention to the distracting sound, leading to failure of the performance of the initial (visual) task (19).

The first aim of this study was to investigate the effect of passive auditory distraction on functional gait in older vs. younger healthy adults. We used the standard Functional Gait Assessment (FGA) (20) and an auditory FGA protocol with an informational type two-talker babble noise masker in a passive listening task for this purpose. Secondary aims were to investigate the relationship between age, hearing capacity, cognitive function (predominantly visual attention allocation) and the individual's functional gait performance in standard and noisy conditions. It was hypothesised that (1) exposure to babble noise would degrade FGA performance in both groups; (2) FGA performance in babble noise conditions would be worse in older compared to younger adults; (3) increasing age, poorer hearing ability and lower performance on cognitive function tasks would be correlated with worse FGA measures.

## Methods

This study was a pilot study and part of a case-controlled study at King's College London, UK in collaboration with University College London, UK. The study was an independent experiment that was conducted within another study at King's College London, UK (Ethical approval Reference LRS-18/19-8994).

### Participants

The study population (*n* = 40) consisted of 20 community-dwelling healthy younger adults (*n* = 20, male = 7; M_age_ 27.0 ± 5.22 years; range 18–35) and 20 healthy older adults (*n* = 20; male = 6; M_age_ 71.5 ± 4.43 years; range 65–80). All participants were screened for compliance with inclusion criteria using a participant-screening questionnaire. Every subject that agreed to participate in the study signed an informed consent.

#### Inclusion Criteria

Eligible participants were healthy young adults aged 18–35 years old or older adults aged 65–80 years old, who lived independently in the community or were independently mobile. All individuals were proficient in written and spoken English.

#### Exclusion Criteria

Individuals were excluded if they had (self-reported): (a) a hearing aid; (b) a diagnosed inner ear disorder which might affect balance performance; (c) an acute limb or other orthopaedic injury that had an effect on balance; (d) neurological conditions such as stroke, epilepsy, peripheral neuropathy or Parkinson's disease that may affect balance and/or walking ability; (e) diagnosis of any cognitive problems such as mild cognitive impairment or dementia.

### Methods

The collected measures of this study were performed in a randomised order. The auditory FGA task was one dual-task that was included as part of a broader study using also other dual-tasks, which were performed in a randomised order. The FGA standard task was always completed first.

#### Functional Gait Assessment (FGA)

The FGA is a standardised test for assessing gait performance (20), during 10 activities including walking with head turns, eyes closed, and stepping over obstacles. Each of the 10 FGA items is scored from zero, for severe impairment, to three for normal performance (21). A cut-off score of 22/30 classifies fall risk and predicts unexplained falls in community-dwelling older adults within 6 months (21). The FGA was performed under two conditions:

1) Standard FGA, completed first, in a silent environment.
2) Auditory FGA, completed second, performed in the presence of an informational noise masker (i.e., noise with an informational content). A multi-speaker babble noise was used since this is the most common environmental background noise where listeners report problems (22). The babble noise masker was a mix of two separate continuous discourses by two independent speakers that were telling a different story. The noise was delivered to the subject's ears at comfortable hearing levels via plastic ear pods, which were connected to a Blu HD 6.0 Android phone. Participants were instructed to perform the FGA while listening to the babble, but were not given any related tasks or asked to listen actively to the babble.

#### Standard Pure Tone Audiometry

Standard pure tone audiometry was conducted at a frequency of 500, 1,000, 2,000, 4,000, and 6,000 Hz to establish a person's hearing threshold level. A four-frequency pure tone average (PTA) of the hearing thresholds was obtained for 500, 1,000, 2,000, and 4,000 Hz for the better ear. Normal hearing is defined as a PTA below 25 dBHL at all frequencies (23). All measurements were performed in a silent room using a portable calibrated audiometer (GSI Pello Standard model with DD45's, IP30, and B81, Serial Number: GS0071085, calibrated by Guymark UK Ltd.).

#### Speech in Babble Test (SiB)

The SiB test is an adaptive, low redundancy speech in babble type noise test, that uses real words as targets, pronounced by a phonetically-trained adult female speaker of Standard Southern British English origin and presented in the background of 20-talker babble noise (22). The test was presented monaurally in a silent room on a calibrated computer using custom-written Matlab software via Sony WIRELESS COMFORT MDR-RF811RK headphones. A signal to noise ratio (SNR) threshold value is calculated as the mean of six to eight reversals, which represents the SNR needed for a performance level of about 50% correct, also known as the Speech Reception Threshold (SRT), which is referred to as SiB score (22).

#### Cambridge Neuropsychological Test Automated Battery (CANTAB)

The CANTAB core cognition battery, that may detect subtle cognitive changes in healthy ageing persons (24), was used to assess neurocognitive function in the healthy younger and older adults. The tests included the Rapid Visual Processing test (RVP), Reaction Time test (RTI), Motor Screening Task (MOT) and Multi-Tasking Test (MTT) (see Table 1), and were conducted in a random order for each participant.

**Table 1:** Explanation CANTAB tests.

**Test name**	**Aim of test**	**Task procedure**
Reaction Time test (RTI)	This test measures a person's speed of response to a visual target when the stimulus is unpredictable.	The participant must start by selecting and holding a button at the bottom of a screen. Hereafter circles were presented above and a yellow dot will appear in one of the circles. The individual had to react as soon as possible, releasing the button at the bottom of the screen, and selecting the circle in which the dot appeared. Outcome measures assessed movement time (ms). Lower scores indicate a better score.
Rapid Visual Processing test (RVP)	This test assesses visual sustained attention.	In the centre of the screen a white box was shown, inside which digits from 2 to 9 appeared in a pseudo-random order, at the rate of 100 digits per minute. Participants needed to detect target sequences of digits and when the target sequence was seen, a response must be given by selecting the button in the centre of the screen as quickly as possible. The level of difficulty varied with either one- or three-target sequences that the participant must watch for at the same time. Outcome measures covered response latency (ms) with lower scores indicating a better score.
Motor Screening Task (MOT)	This test provides a general assessment of whether sensorimotor deficits or lack of comprehension will limit the collection of valid data from the participant.	In this task, coloured crosses were presented in different locations on the screen, one at a time. The participant had to select the cross on the screen as quickly and accurately as possible. Outcome measures assessed the individual's speed of response (ms). Lower scores indicate a better performance.
Multi-Tasking Test (MTT)	This test assesses the participant's ability to manage conflicting information and to ignore task-relevant information.	The test displays an arrow, which can appear on either side of the screen and can point in either direction. A cue is displayed at the top of the screen that indicates whether the individual needs to select the right or the left button according to the side of the arrow's appearance or the direction in which the arrow was pointing. Using both rules in a flexible manner places a higher demand on cognition than using a single rule since the rule is changed from trial to trial in a randomised manner. Outcome measures indicated the multitasking cost (ms). A positive score indicates a higher cost (i.e., slower response during multitasking).

The CANTAB was administered on a handheld tablet in a quiet room. The subject was sitting in a comfortable position with the screen at a 0.50 m distance at their eye level. The test administrator sat next to the subject and the subject used the index finger of their dominant hand to touch the screen of the tablet. Auditory and visual instructions were given via the CANTAB itself. When necessary the test administrator could give again the exact instructions. There was the opportunity to rest after each test, if desired.

### Statistical Analysis

Statistical analysis was executed using IBM SPSS statistics 25 software for Mac OS X (Armonk, NY). Statistical significance was set at *p* < 0.05 for all computations. Data was checked for normality of distribution by the Shapiro-Wilk test and presented as mean ± Standard Deviation (SD). Mann-Whitney *U*-tests were run to evaluate significant differences between the two age groups for all variables. A Wilcoxon rank test was used to determine a possible significant difference between FGA standard and FGA audio in general. The dual-task cost (DTC), i.e., the percentage change in FGA performance due to the dual-task condition, was calculated for standard FGA vs. auditory FGA using the following equation (25):

$$\begin{array}{l} \text{Dual task cost }\left(\text{\%}\right)=100\;*\;\left(\frac{\text{Multi task}-\text{Single task}}{\text{Single task}}\right) \end{array}$$

Negative DTC values indicate a decrease in FGA score, which means a worse task performance in dual-task condition.

Furthermore, a Spearman correlation matrix was generated using R studio (version 4.0.3; Boston, MA) to assess possible associations among age, PTA scores, SiB scores, MTT scores, RTI scores, RVP scores, FGA standard scores, FGA audio scores, and FGA dual-task cost. *P*-values were adjusted according to Holm correction.

## Results

A summary of the descriptive statistics of audiological, cognitive and functional gait test results in the young and old participants is reported (see Table 2).

**Table 2:** Descriptive statistics of the various variables.

**Characteristics**	**Young**	**Old**	**Mean difference**	***P*-value**
	**Mean ± SD**	**Mean ± SD**		
**Number of participants (*****n*****)**	20	20		
**Age (years)**	27.0 ± 5.22	71.5 ± 4.43	44.5	0.000
**Gender**				
Male	7	6		
Female	13	14		
**PTA average, better ear (dBHL)**	11.3 ± 3.43	22.2 ± 7.00	10.9	0.000
>25	0	5		
= <25	20	15		
**SiB average, better ear (dB)**	0.18 ± 1.79	2.22 ± 2.02	2.04	0.001
>3.5	1	5		
= <3.5	19	15		
**CANTAB subtest (ms)**				
MOT	692 ± 161	819 ± 130	127	0.005
MTT	167 ± 117	287 ± 145	120	0.001
RTI	232 ± 54.3	295 ± 70.2	63	0.003
RVP	437 ± 78.6	510 ± 66.8	73	0.001
**FGA (/30)**				
**Standard**	29.3 ± 0.72	26.6 ± 2.04	2.70	0.000
>22/30	20	20		
= <22/30	0	0		
**Auditory**	28.9 ± 0.99	25.8 ± 2.22	3.10	0.000
>22/30	20	18		
= <22/30	0	2		

*PTA, Pure Tone Average; dBHL, Decibel Hearing Loss; SiB, Speech in Babble test; CANTAB, Cambridge Neuropsychological Test Automated Battery; MOT, Motor Screening Task; MTT, Multi-Tasking Test; RTI, Reaction Time test; RVP, Rapid Visual Processing test; FGA, Functional Gait Assessment*.

**Figure 1:**
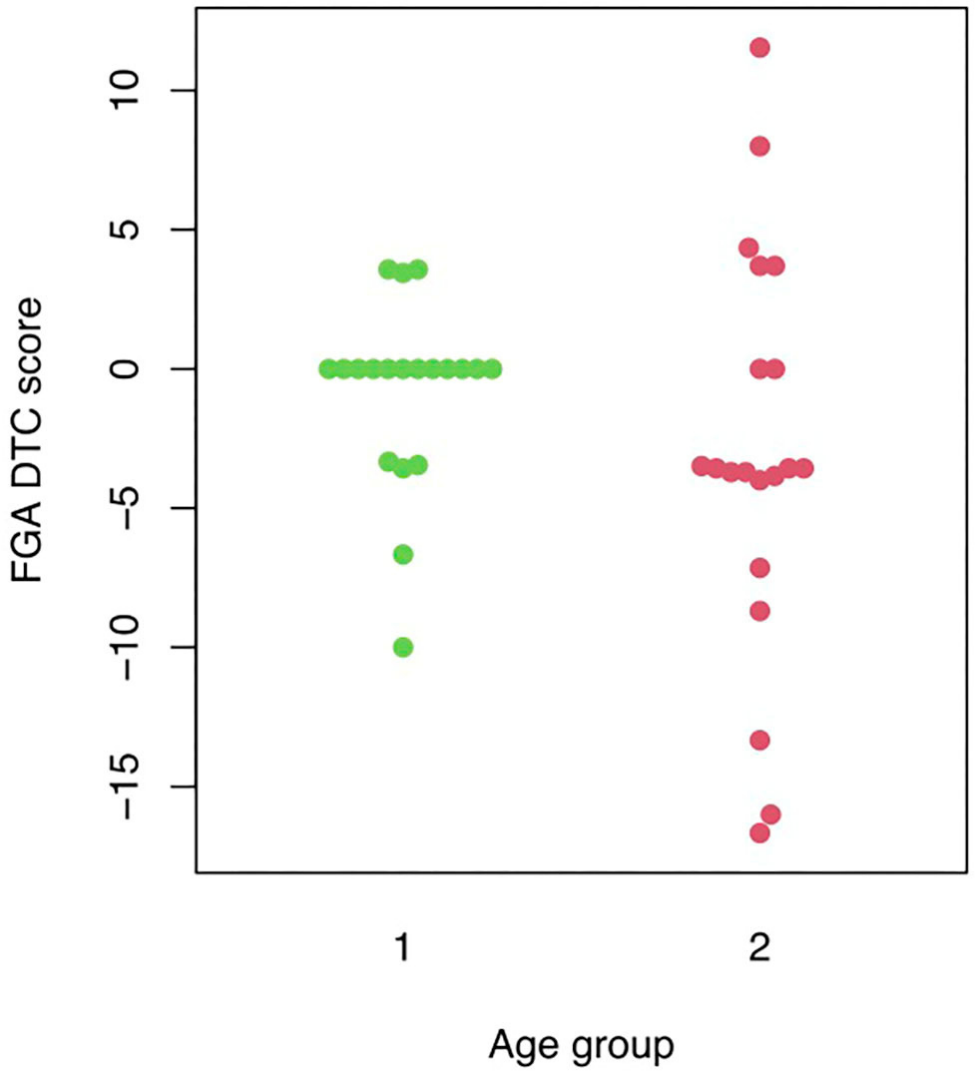
Visual representation of the individual FGA auditory dual-task cost scores in the two different age groups. FGA DTC, Functional Gait Assessment dual-task cost; 1, young age group; 2, old age group.

Mann-Whitney *U*-tests were run to determine possible FGA differences between the two age groups. Statistical analysis showed: (1) a significant difference between the two age groups for FGA standard scores (*p* = 0.000); (2) FGA audio performance differed significantly between the two age groups (*p* = 0.000); (3) no significant difference between young and old adults for their mean FGA dual-task cost values (*p* = 0.11) (see Figure 1). Wilcoxon tests indicated that FGA in standard condition did not differ significantly from FGA in auditory condition in both the young (*p* = 0.14) and the old age group (*p* = 0.077). Because of this finding, the age groups were collapsed and a Wilcoxon test was performed to assess the difference in FGA standard and FGA auditory scores. A significant difference with a mean dual-task cost of −1.91% (SD = 5.69; *p* = 0.022) was found between FGA standard and FGA auditory performance in the combined group.

### Spearman Correlation

A negative correlation was found between age and FGA standard scores (*r* = −0.73; *p* = 0.000; Holm *p* = 0.00), and between age and FGA audio scores (*r* = −0.63; *p* = 0.000; Holm *p* = 0.00). This indicates that with increasing age, the FGA standard and FGA auditory scores are likely to decrease (i.e., worse performance). Positive correlations were found between age and PTA (*r* = 0.69; *p* = 0.000; Holm *p* = 0.00), and SiB scores (*r* = 0.45; *p* = 0.004; Holm *p* = 0.10). Furthermore, correlations were observed for MTT scores and age (*r* = 0.36; *p* = 0.024; Holm *p* = 0.44), PTA (*r* = 0.65; *p* = 0.000; Holm *p* = 0.00), SiB (*r* = 0.53; *p* = 0.000; Holm *p* = 0.01), RTI (*r* = 0.46; *p* = 0.003; Holm *p* = 0.08), RVP (*r* = 0.46; *p* = 0.003; Holm *p* = 0.07), and FGA audio (*r* = −0.40; *p* = 0.011; Holm *p* = 0.24) scores. Positive correlations between RVP and age (*r* = 0.44; *p* = 0.004; Holm *p* = 0.10), PTA (*r* = 0.47; *p* = 0.002; Holm *p* = 0.07), SiB (*r* = 0.41; *p* = 0.008; Holm *p* = 0.17), RTI (*r* = 0.63; *p* = 0.000; Holm *p* = 0.00), and a negative correlation with FGA audio (*r* = −0.49; *p* = 0.001; Holm *p* = 0.04) were indicated. Interestingly, higher visual processing response latency scores (i.e., worse performance) are significantly associated with lower FGA auditory scores. Correlations were found between RTI and age (*r* = 0.47; *p* = 0.002; Holm *p* = 0.07), PTA (*r* = 0.47; *p* = 0.002; Holm *p* = 0.07), FGA audio (*r* = −0.50; *p* = 0.001; Holm *p* = 0.03), and FGA DTC (*r* = −0.42; *p* = 0.007; Holm *p* = 0.15) scores. Longer movement times are significantly associated with lower FGA audio scores (i.e., worse performance). Finally, PTA scores correlated negatively with FGA standard (*r* = −0.58; *p* = 0.000; Holm *p* = 0.00), and FGA audio scores (*r* = −0.67; *p* = 0.000; Holm *p* = 0.00). This indicates that higher scores (i.e., worse hearing capacity) are significantly associated with lower FGA scores (i.e., worse performance). Negative correlations were also indicated between SiB scores and FGA standard (*r* = −0.34; *p* = *p* = 0.030; Holm *p* = 0.51), FGA audio (*r* = −0.52; *p* = 0.001; Holm *p* = 0.02), and FGA DTC scores (*r* = −0.34; *p* = 0.034; Holm *p* = 0.54).

## Discussion

To our knowledge, this is the first study to assess functional gait while performing a passive listening task. As hypothesised, the exposure to babble noise degraded FGA performance in both groups, regardless of an individual's age. This finding may be of clinical relevance, in that this passive auditory distraction puts two out of 20 healthy older adults at risk of falls (FGA <22) (21). However, this change in FGA score may have a smaller impact than statistically suggested. The results must therefore be interpreted carefully as changes on the FGA should perhaps be more pronounced to be clinically meaningful. Nevertheless, such exposure to passive listening while walking is happening on a daily basis (17). The impact of this situation needs to be further investigated in healthy older adults as well as in adults at risk of falls to address the need for more optimal rehabilitation approaches.

According to the “cognitive load” hypothesis, a reduction in balance performance in the presence of a concurrent cognitive task indicates a decreased amount of attentional capacity [(26) for a review (27)]. This is attributed to the secondary cognitive task acting as a distractor receiving attentional capacity, leaving less attentional capacity available for balance control [(28) for review (11, 29, 30)]. Most studies using healthy adults report that the performance of a secondary task influences gait (16, 29, 31, 32), and that even healthy young adults seem to generally walk more slowly in dual-task conditions (15). A substantial body of evidence indicates that gait, even in young healthy adults, utilises attention [(32, 33) for a review (34)].

The simultaneous performance of two attention-demanding tasks not only cause competition for attention, it also challenges the brain to prioritise the two tasks [(34) for a review]. Several studies report that both young and older healthy adults maintain gait stability when walking and performing a cognitive task, but with a decline in the cognitive task performance (1, 11, 35, 36), i.e., they show a “posture first” strategy (26, 37). This may occur because attentional capacity decreases with age, and older adults tend to prioritise their dynamic stability to avoid falling [(28) for a review; (27) for a review]. However, Liston and colleagues reported that older adults may fail to prioritise postural tasks when dual-tasking, indicating a deviation from the posture first strategy (25). In their study a bi-modal spatial multi-task test was performed consisting of a visually-coded spatial step navigation task and an auditory-coded spatial congruency task. Healthy older adults prioritised temporally regular cognitive tasks rather than the postural task. Nevertheless, in our study, this posture first strategy might potentially account for the overall rather small negative dual-tasking effect due to the passive auditory distraction. Since the adults might have prioritised their gait, most of their attention was potentially used to perform the balance tasks instead of listening to the informational masker. In line with this, no differential impact of ageing on the interference between postural and cognitive processing, using a simple auditory reaction time task during rapid destabilising floor translation, was also reported by Muller et al. (33). Furthermore, there was no effect of auditory challenge on postural measures found for either young or old adults, as well as old adults with age-related hearing loss in the study of Bruce et al. (14).

The concurrent cognitive task in this study was a passive listening task, with the participants exposed to a speech discourse without being asked to listen or follow the story, or asked questions. The participants could thus opt to ignore this input. Nevertheless, it was sufficient to significantly reduce the efficiency of balance in the study population, regardless of the participant's age. Beaman and colleagues suggested that even unattended speech engages attention networks and reported a disruptive effect of supposedly unattended sound on cognitive functioning (38). The presence of task-irrelevant background speech can increase the error rate on a primary task, such as memory performance (39), possibly by taking up cognitive resources that would otherwise be available for other tasks. This would be consistent with the “cognitive load” hypothesis (38).

Despite a slightly larger dual-task cost score for the older adult group, there was no significant difference in the dual-task costs between elderly and young adults. This may be due to the relatively low challenge of the dual task. Older adults appear to be particularly vulnerable to auditory distraction by irrelevant stimuli when performing a range of tasks and show an increased activity in the default mode regions compared to younger adults, in whom specific regions are deactivated to be able to encode successfully the target stimuli (19, 40). However, the default mode regions were equally deactivated in both age groups in another imaging study that used a simple repetition-priming task (41). This lack of age effect was attributed to the fact that the task was relatively easy for both young and older participants (41). The same could account for the non-significant difference in dual-task costs between the two test groups in the present study.

Our third hypothesis stated that there would be associations between age, hearing ability, cognitive function, and FGA measures. A few remarkable correlations were found. Age was associated with all measures, except for FGA DTC. This indicates that ageing is indeed associated with a decline in balance, hearing, and cognitive function. However, after Holm correction only a trend was considered between age and some cognitive measures. These non-significant correlations could be due to the under powering of the study. Positive significant correlations were observed between hearing capacity and all cognitive measures, although part of these were considered a trend or not significant after correction. Some significant negative correlations were found between hearing capacity and FGA measures. This means that a worse hearing capacity results in a decrease in some of these cognitive and functional gait performances. Furthermore, significant negative correlations were observed between visual reaction processing scores and FGA performances, and between RTI subtest movement times and FGA audio scores. Taken together, this implies that a worse performance on attention tasks seems to be associated with a worse FGA performance in the noisy condition. The role of cognitive factors, especially attention, in the control of balance is evident during both standing and walking [(1, 32) for a review (27)]. People with less available attentional resources are likely to experience difficulties with postural tasks when two or more tasks require those cognitive resources. Interestingly, a negative influence of tinnitus, i.e., the internal percept of sounds that do not arise from external sources, on executive cognitive control (e.g., some working memory scores, attention tasks) has been indicated in the literature (42, 43). Patients experiencing higher subjective tinnitus suffer from a higher burden on attentional resources (42). In this study, passive listening to multi-talker babble noise possibly occupied some of the attentional resources, which challenged the execution of the postural tasks.

Limitations of this study need to be acknowledged. First of all, the study comprised a rather small sample size and the correlation analysis in particular should be repeated with a bigger sample. The healthy older adults group was not very representative of the general older adult population as they were relatively fit. Furthermore, some observer bias needs to be taken into account for judging the FGA task, and also a physical and mental fatigue should be considered as the complete study lasted 2 h. There was no matching for educational level. Finally, it was not asked if the participants ignored the story or actively listened to it. The noise was moreover delivered through ear pods that masked the spatial aspect. Since this spatial element of environmental sound can induce localisation, a delivery without the usage of ear pods might have an even more negative effect on FGA scores. Future experiments should take these considerations into account. However, this is the first study to assess and find the degrading effect of a passive listening task on an individual's functional gait performance.

## Conclusion

A similar negative dual-task effect was observed in both age groups for a low demand, passive two-talker listening task. This irrelevant sound can take up cognitive resources that could induce a cognitive load, which then would result into disruptive effects on the performance of the balance task. Nevertheless, the results should be considered preliminary as this is a pilot study. Therefore, further research is needed using a larger sample size that represents a more realistic measure of elderly suffering from age-related declines, to support these preliminary results. Furthermore, a more challenging concurrent auditory or cognitive task may be incorporated in future studies to investigate potentially more pronounced dual-task costs. Brain imaging might be a helpful method to further investigate the hypothesis of auditory distraction and its cognitive load due to activated neural networks, even in unattended speech/noise conditions.

## Data Availability Statement

The raw data supporting the conclusions of this article will be made available by the authors, without undue reservation.

## Ethics Statement

The studies involving human participants were reviewed and approved by Biomedical Sciences, Dentistry, Medicine and Mathematical Sciences Research Ethics Subcommittee, King's College London, UK. Ethical Approval Reference: LRS-18/19-8994. The patients/participants provided their written informed consent to participate in this study.

## Author Contributions

D-EB and MP designed the study and study paradigms. MB and VA conducted the tests. MB analysed the results and drafted the paper. All authors reviewed and contributed to the analysis, the paper, and approved the final manuscript.

## Conflict of Interest

The authors declare that the research was conducted in the absence of any commercial or financial relationships that could be construed as a potential conflict of interest.

## Abbreviations

CANTAB: Cambridge Neuropsychological Test Automated Battery
dB: Decibel
dBHL: Decibel Hearing Loss
DTC: Dual-Task Cost
FGA: Functional Gait Assessment
MOT: Motor Screening Task
MTT: Multi-Tasking Test
PTA: Pure Tone Average
RTI: Reaction Time test
RVP: Rapid Visual Processing test
SiB: Speech in Babble test
SNR: Signal to Noise Ratio
SRT: Speech Reception Threshold.
